# Upper cervical spine injuries: a management of a series of 70 cases

**DOI:** 10.11604/pamj.2013.15.57.2316

**Published:** 2013-06-20

**Authors:** El Fatemi Nizare, Bouchaouch Abdelali, Derkaoui Hassani Fahd, Oudrhiri Mohammed Yassad, Gana Rachid, El Maaqili Rachid, Bellakhdar Fouad

**Affiliations:** 1 Service de Neurochirugie, Hôpital Ibn Sina, CHU Rabat, Faculté de Médecine et de Pharmacie de Rabat, Université Mohammed V – Souissi, Maroc

**Keywords:** Upper cervical spine, injury, surgical management, prognosis

## Abstract

Traumatic injuries of the upper cervical spine are often encountered, and may be associated to severe neurological outcome. This is a retrospective study of 70 patients, admitted over a 14 years period (1996 to 2010), for management of upper cervical spine injuries. Data concerning epidemiology, radiopathology and treatment was reviewed, and clinical and radiological evaluation was conducted. Men are more affected than women, with traffic accidents being the major traumatic cause. A cervical spine syndrome of varied intensity was found in about 90% of patients; neurological deficit was noted in 10 patients (21%). Radiological analysis discovered varied and many combined lesions: C1-C2 dislocation (7 cases), C2-C3 dislocation (9 cases), C1 fracture (10 cases) and C2 fracture (44 cases) including 28 odontoid fractures. Orthopedic treatment was carried out exclusively for 31 patients, and surgical treatment for 38 patients. One patient died before surgery because of a polytraumatisme. Posterior approach was performed in 29 cases including hooks and rods in 18 patients, wiring in 9 cases, and 2 transarticular screw fixations. In 9 cases anterior approach was performed: 5 odontoid screwing and 4 cases of C2-C3 discectomy with bone graft. Nearly all patients were improved in post-operative. Elsewhere, the operating results were marked by a persistent neurological deficit in 2 cases, and infection in 2 cases controlled by medical treatment. Mean follow-up was 23 months and showed good clinical and radiological improvement. Early management of cervical spine injuries can optimize outcome. Treatment modalities are well codified; however controversy remains especially with type II odontoid fractures.

## Introduction

Upper cervical spine injuries are frequent due to increasing number of road accidents and falls. These lesions are often serious but neurologic complications are rare in emergency; this is due to the fact that C1-C2 medullar injuries are fatal. In survivors, those lesions may cause chronic instability with pseudarthrosis or delayed neurologic complications. Consequently, every trauma patient needs X-ray and adequate treatment. Authors establish therapeutic indications of upper cervical spine injuries according to clinical and radiological information and assess long term prognosis of instability and neurological injuries.

## Methods

This is a retrospective study of patients with upper cervical spine injuries managed at the neurosurgical department in IBN SINA hospital at Rabat between 1996 and 2010. We also included patients who had other lesions in polytrauma context. Neurological deficit was assessed by Frankel scale. Imaging was used to classify the types of fracture. The treatment has included orthopedic and surgical methods. The rates of fusion and neurological recovery were analyzed long-term.

## Results

70 cases were included in the study. Young adults were concerned in 66% with range from 16 to 65 years. Sex ratio (M/F) was 4. Road accidents were the most frequent cause with often hyperflexion mechanism. Clinically, cervical spine syndrome was found in 90% of cases, neurological deficit was present in 14 cases (21%). Most patients were admitted with 24 hours delay (83%). Disorders of consciousness concerned 7 cases of serious polytrauma. Radiological analysis included standard X-ray and CT scan with Multiplanar reconstruction in all patients. MRI was performed in 11 cases. Radiological lesions are various and sometimes associated as shown in Table 1.

**Table 1:** Radiological lesions in cervical CT-san

C1-C2 dislocations	7 cases
C2-C3 dislocations	9 cases
C1 fractures	10 cases
C2 fractures	44 cases (28 odontoid fractures; 8 hangman's fractures; 8 C2 pedicle fractures)

A lower cervical spine injury was associated in 11 cases, and 20 upper cervical spine injuries were diagnosed in a polytrauma. One patient with C2-C3 dislocation died from hemodynamic instability before surgery. Patients with neurological deficits admitted in 6 hours delay had corticosteroids. A transcranial traction was performed in emergency in all cases to avoid secondary displacement. Reduction was obtained in all cases. It was incomplete in 5 cases (4 C2-C3 dislocations and a C1-C2 dislocation).

Orthopedic treatment was performed in 31 cases of stable fractures using Minerva with triple support (Table 2). 38 patients were operated on. 29 cases with posterior approach including hooks and rods in 18 patients, wiring in 9 cases, and 2 transarticular screw fixation. In 9 cases anterior approach was performed: 5 odontoid screwing and 4 cases of C2-C3 discectomy with bone graft. Neurologic improvement was found in 74% cases postoperatively without post-operative death. The fusion rate was 94% in operated patients along a mean period of 23 months (Table 3). Orthopedic treatment provides pseudarthrosis in 22% of cases with neurologic improvement in 93% of cases.

**Table 2:** Orthopedic treatment

C1 fractures	Ring fractures	3
	Lateral mass fracture	3
C1-C2 fractures	Ring fractures; Hangman's type I fracture	4
C2 fractures	Hangman's type I fracture	3
	Body non displaced fractures	4
	Type I odontoid fractures	2
	Type III odontoid fractures	4
	Type II oblique fractures	5
	Type II horizontal fractures	3

**Table 3:** Post-operative evolution

		Clinical results	Number	%
Posterior approach	Hooks and rods	Improvement	10	55.5
		Cervical pain	3	16.6
		Stiffness of the spine	5	27.7
	Wiring	Improvement	5	55.5
		Stiffness of the spine	3	33.3
		pseudarthrosis	1	11.1
	Transaricular screw fixation	Improvement	2	100
Anterior approach	Odontoid screwing	Improvement	5	100
	C2-C3 discectomy	Improvement	4	100

## Discussion

In our series as in those of the literature, upper cervical spine injuries concerns young male population [1–4]. C0-C1-C2 dislocations in upper cervical spine injuries are rare and fatal. On survivors, neurological deficit is minimal or absent so, those lesions may not be diagnosed especially on polytrauma or coma patients causing instability with risk of sudden severe neurologic impairment. Thus, a complete radiologic examination is needed in emergency. It includes standard X-ray, 3D CT-scan and sometimes cervical MRI depending on lesions and clinical data especially in coma patients as recommended by Robert et al [5]. It leads to a detailed assessment of lesions, the choice of therapeutic approach, and the post-operative follow-up in terms of fusion rate and detecting pseudarthrosis. In our department, we use the Roy Camille Classification of Odontoid fracture. Most frequent lesions are those of C2 (44/70 in our series), especially concerning odontoid (28/44); those lesions are often unstable and needs surgical fixation as described in literature [6–9].

**Figure 1:**
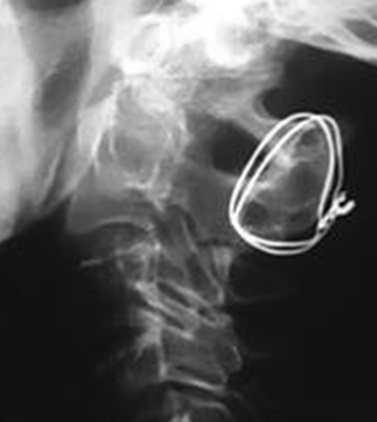
Post operative standard X-ray showing a patient who benefited from wiring

**Figure 2:**
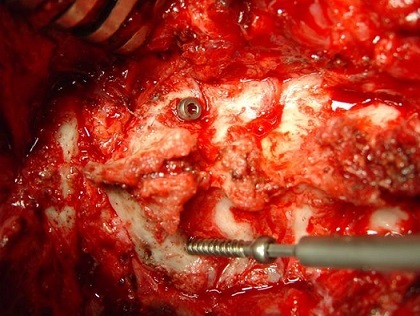
Intraoperative view showing a case of transarticular C1C2 screwing

**Figure 3:**
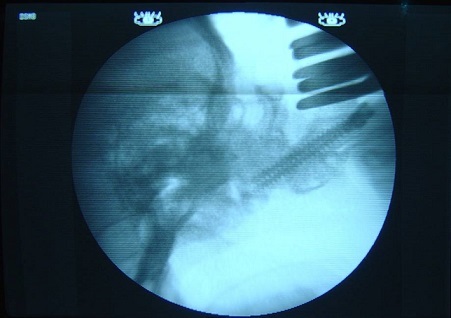
Intraoperative view showing fluoroscopic control of transarticular C1C2 screwing

**Figure 4:**
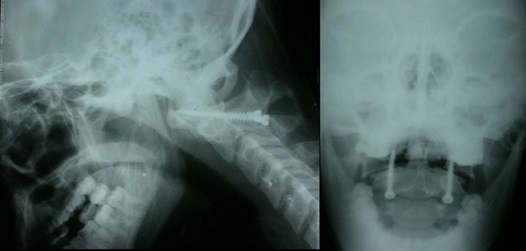
Post operative standard X-ray showing a patient who underwent transarticular screwing

**Figure 5:**
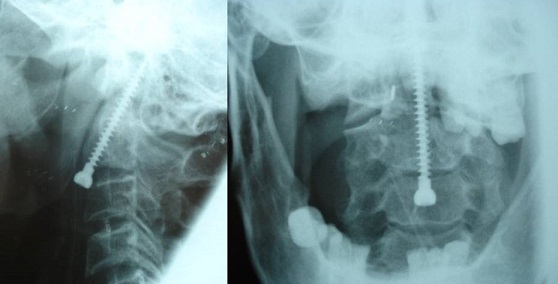
Post operative standard X-ray showing a patient who benefited from odontoid screwing

**Figure 6:**
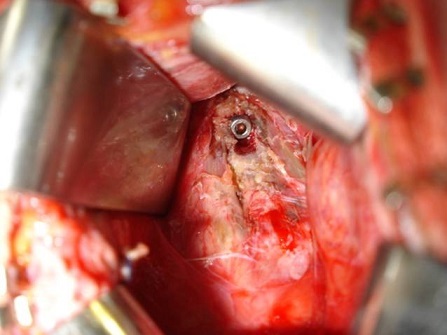
Intraoperative view showing a case of anterior odontoid screwing

**Figure 7:**
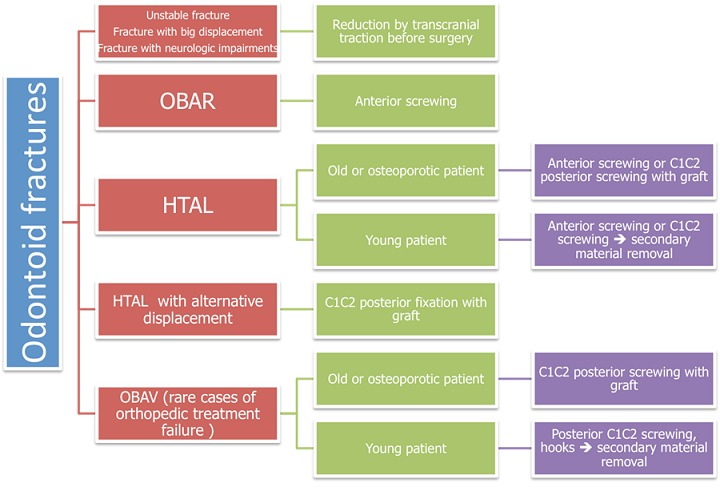
Therapeutic algorithm of odontoid fractures

The choice of the surgical technique depends on the location of the fracture, the displacement, associated lesions and the bone quality. Reduction of displacements is essential even pre or post operatively. Operative delay depends on clinical status, neurologic examination, general and cervical assessment of lesions. Less than 3 levels osteosynthesis is preferred to respect the biomechanics of the cervical spine; associated to a fusion, it avoids the wear of the material. In odontoid fractures, there are lots of surgical techniques [9]: posterior wiring and bone grafting (Figure 1), transarticular screw fixation (Figure 2, Figure 3, Figure 4, Figure 5), screws in the lateral mass of C1, odontoid screw fixation (Figure 6, Figure 7). We prefer to use odontoid screwing which allow us to respect the biomechanics of the cervical spine.

Anterior screwing contraindications of the odontoid are: anatomic irreducibility, pseudarthrosis, OBAV fracture, major osteoporosis, important comminution of the odontoid basis, an associated fracture dislocation of C1, a ruptured transverse ligament, pathologic fracture, severe kyphosis of cervical spine, short neck and protruding sternum. Post operative results are: 69% fusion, 24% secondary displacement due to technical mistake, and 7% pseudarthrosis. The mean fusion rate reported by Julien according to some retrospective studies was 89% in type II odontoid fractures [10–16].

Magerl's posterior transarticular screwing of C1C2 [17] is indicated in case of type II unstable OBAR fracture with C1C2 dislocation, Jefferson's fracture, type II unstable OBAV fracture, odontoid pseudarthrosis (in case of C1C2 wiring). Isolated screwing is preferred in case of transitory traumatic instability; its contraindications are: aberrant course of vertebral artery, narrow isthmus of C2, bone lesions in the site of screwing, bad visualization of C1C2 on scope. Definitive fusion is obtained in 95% to 98% of cases [18].

C0-C1-C2-C3 posterior fusion by hooks and rods may lead to C1 posterior arc fracture and fusion failure of the interbody graft in 10 to 25% of cases. 44.3% of our patients were managed by orthopedic treatment using made to measure minerva. Most of them interested C2 and were stable fractures. Some authors [19, 20] use the halo vests to manage upper cervical spine fractures; its place is well defined; however, it is more cluttering and uncomfortable for the patient; it also takes more time for fracture healing and expose to the risk of pseudarthrosis. Surgical treatment will be indicated in this case.

## Conclusion

Clinico-radiological analysis of upper cervical spine injuries leads to better therapeutic indications and improves long-term outcome in terms of spine stability and biomechanics; thus fusion is needed in every unstable lesion, because orthopedic treatment alone leads to pseudarthrosis in many cases.
